# Two-Stage Processing of Sounds Explains Behavioral Performance Variations due to Changes in Stimulus Contrast and Selective Attention: An MEG Study

**DOI:** 10.1371/journal.pone.0046872

**Published:** 2012-10-11

**Authors:** Jaakko Kauramäki, Iiro P. Jääskeläinen, Jarno L. Hänninen, Toni Auranen, Aapo Nummenmaa, Jouko Lampinen, Mikko Sams

**Affiliations:** 1 Department of Biomedical Engineering and Computational Science (BECS), Brain and Mind Laboratory, Aalto University School of Science, Espoo, Finland; 2 Advanced Magnetic Imaging Centre, O.V. Lounasmaa Laboratory, Aalto University School of Science, Espoo, Finland; 3 Brain Research Unit, O.V. Lounasmaa Laboratory, Aalto University School of Science, Espoo, Finland; 4 Athinoula A. Martinos Center in Biomedical Imaging and Department of Radiology, Massachusetts General Hospital, Charlestown, Massachusetts, United States of America; National University of Singapore, Singapore

## Abstract

Selectively attending to task-relevant sounds whilst ignoring background noise is one of the most amazing feats performed by the human brain. Here, we studied the underlying neural mechanisms by recording magnetoencephalographic (MEG) responses of 14 healthy human subjects while they performed a near-threshold auditory discrimination task vs. a visual control task of similar difficulty. The auditory stimuli consisted of notch-filtered continuous noise masker sounds, and of 1020-Hz target tones occasionally (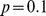) replacing 1000-Hz standard tones of 300-ms duration that were embedded at the center of the notches, the widths of which were parametrically varied. As a control for masker effects, tone-evoked responses were additionally recorded without masker sound. Selective attention to tones significantly increased the amplitude of the onset M100 response at 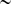
100 ms to the standard tones during presence of the masker sounds especially with notches narrower than the critical band. Further, attention modulated sustained response most clearly at 300–400 ms time range from sound onset, with narrower notches than in case of the M100, thus selectively reducing the masker-induced suppression of the tone-evoked response. Our results show evidence of a multiple-stage filtering mechanism of sensory input in the human auditory cortex: 1) one at early (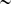
100 ms) latencies bilaterally in posterior parts of the secondary auditory areas, and 2) adaptive filtering of attended sounds from task-irrelevant background masker at longer latency (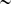
300 ms) in more medial auditory cortical regions, predominantly in the left hemisphere, enhancing processing of near-threshold sounds.

## Introduction

Selective attention has been shown to increase the “gain” of neural responses (i.e., augment responses to attended and suppress responses to non-attended stimuli) both in auditory [1]–[4] and visual [5] modalities. Using adaptation paradigms, magnetoencephalography (MEG) [6], [7] and functional magnetic resonance imaging (fMRI) [8] studies have extended these results by suggesting that, besides the “gain increase” [9], feature selectivity of sensory cortical neurons also increases when attending to sound features. Specifically, neuronal receptive fields are reshaped to match the relevant features of the to-be-attended stimuli. Furthermore, recent findings in electroencephalography EEG [10] and MEG [11] studies suggest that the mechanisms for attentional enhancement and inhibition are distinct. Together, these attentional mechanisms rapidly and task-specifically reorganize human auditory system function (for a review, see e.g. [12], [13]).

Sometimes the “gain” and “tuning” models of selective attention have been viewed as mutually exclusive, however, noise-normalization models of attention have been recently proposed for visual cortex neurons that combines these two views [14], [15]. Specifically, the noise-normalization model proposes that the changes in receptive field shape occur when multiple objects fall within a single-neuron receptive field, or when a neuron's preferred contrast does not match the presented stimulus. An auditory analogue of this is simultaneous occurrence of multiple sounds within the critical band of the attended sound frequency.

**Figure 1:**
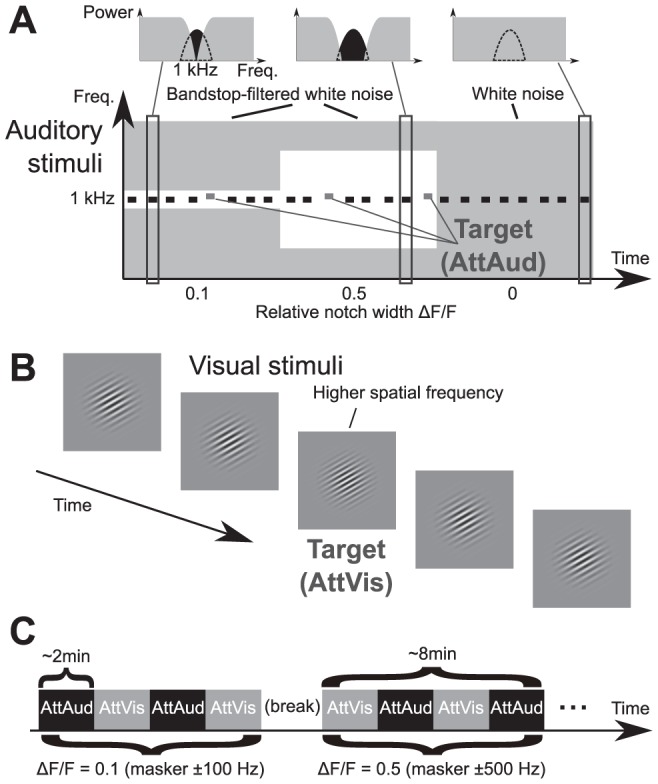
A schematic illustration of the paradigm. (A) During selective auditory attention (AttAud condition), subjects attempted to detect higher-frequency target tones. The background grey represents the noise masker, while the white area represents the frequency gaps in the noise. (B) During visual attention (AttVis condition), target stimuli with higher spatial frequency were to be detected. Auditory and visual stimuli were identical in both conditions. (C) Each masker type was presented for about 8 minutes, with AttAud and AttVis conditions alternating every 2 minutes, indicated by instructions on the screen during the condition start and change.

In humans, auditory-evoked responses are suppressed in amplitude by masking [16], [17]. The amplitude of the N100 response, peaking at around 100 ms after sound onset, increases as the distance in sound frequency increases between the masker and test sounds. Importantly, this effect has been used to derive estimates of underlying neuronal receptive field selectivity (i.e., “frequency tuning” of the neurons). Specifically, it is assumed that neurons with receptive fields that are sharply tuned to sound frequency are minimally affected by notched-noise masker sounds when the notch is wide enough to fall on the neuronal receptive fields. In this case the neurons are left unadapted and thus elicit robust response upon presentation of the test sound. However, as the notch width is narrowed down, the noise masker edges begin to fall on the receptive fields of the neurons that respond to the test sound. This results in adaptation of the neurons and diminution of the response that is generated when the test sound is presented (this is schematically illustrated in the upper panel of Figure 1). Selective attention effects on neuronal receptive fields can then be assessed by studying whether there are changes in the suppressive effects of maskers as a function of decreasing notch width of the maskers.

Tone-in-noise experiments using continuous maskers have suggested a combination of attention-dependent gain and selectivity increase of neural responses using EEG [18] and MEG [7], [19], [20]. Kauramäki and colleagues [18] showed a robust N100 enhancement in EEG while attending to sound compared to a passive silent movie baseline. However, the attentional enhancement was not constant with different masker notch widths, but rather the enhancement in the attend vs. ignore conditions was largest with some of the intermediate notch widths below the critical band, smaller with the widest notches (easiest-to-detect targets) as well as with the white noise masker (hardest-to-detect targets). Thus, these results could not be explained by a simple gain increase alone (i.e., multiplicative increase in the tuning function), but likely involved enhanced selectivity of the receptive fields of underlying neural populations as well. Unfortunately, the limited spatial resolution in this EEG study prevented estimation of the cortical loci of these selective attention effects.

Here, we explored the attention-related frequency tuning changes in the auditory cortex with continuous notched-noise maskers (Fig. 1A). We specifically hypothesized that 1) the biggest attention effects would be seen at intermediate masker notches, 2) exploiting the spatial accuracy of MRI-constrained MEG [21], [22] combined with an extensive selection of masker notches that the effects are localized in the auditory cortex, and 3) we further explored whether similar effects can be seen in the sustained response or whether the tuning effects are unique to M100 latency. Further, we designed the experiment so that possible arousal-related effects were controlled by a visual control task of similar difficulty (Fig. 1B) and so that the build-up of the selective attention effects as a function of time on the task could be assessed by using task switching (Fig. 1C) that requires high level of cognitive control.

## Results

The main findings of the present study were the following: 1) The M100 response peak amplitudes increased and latencies shortened with increasing notch width. 2) Selective attention to sounds increased the M100 response amplitude in both hemispheres with all noise maskers. 3) Attention enhanced the sustained response, localized more medially than M100, selectively with narrow notches especially in the left hemisphere.

### Equivalent current dipole fitting results

**Figure 2:**
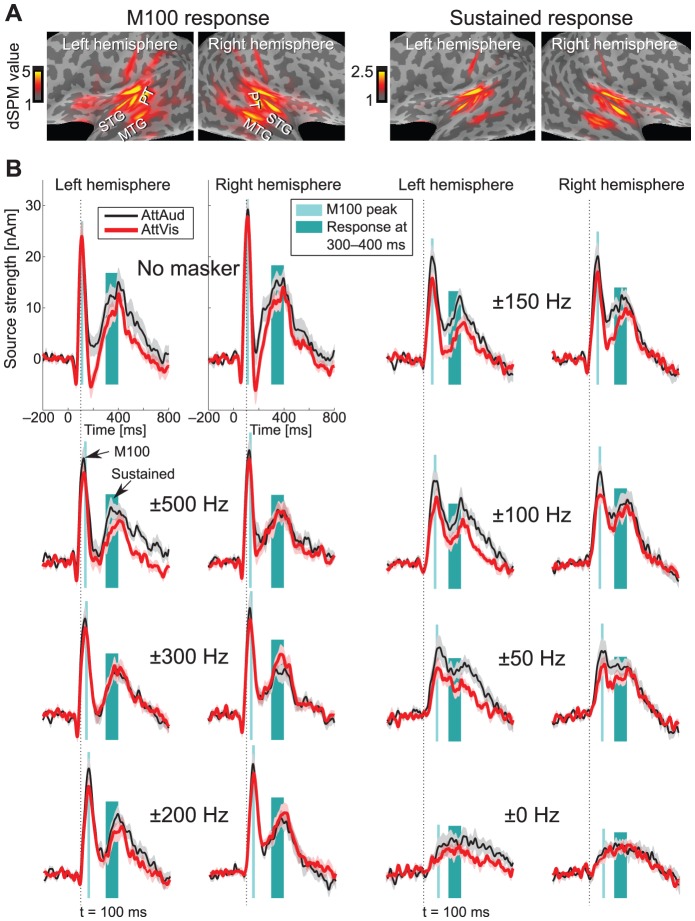
Response suppression due to masking. (A) Grand average (N = 10) dSPM snapshots of activation with AttAud condition. These illustrate the M100 (latency 123 ms) and sustained response activity (mean of latency range 300–400 ms) during playback of the masker with widest notch (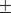
500 Hz). Activations are shown on an inflated cortical surface, darker gray areas corresponding to sulci and lighter areas to gyri. The sustained response shows less background ripple due to averaging of data which in turn increases the signal-to-noise ratio. Note different scales for M100 and sustained response. (B) Grand average (N = 14) source waveforms (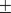
standard error of the mean, SEM, shaded areas) for each stimulus type, hemisphere and condition, projected through the equivalent current dipoles best explaining M100 responses. The source waveforms illustrate the gradual suppression of response amplitudes and increase in latency with narrower notches. Attentional enhancement of sustained response especially in the left hemisphere is evident. Note that for visualization, source waveforms for sustained responses shown here are projected through M100 dipoles which do not exactly coincide with sustained response activity, but the actual analyses use the more anterior and inferior sustained response dipole.

**Figure 3:**
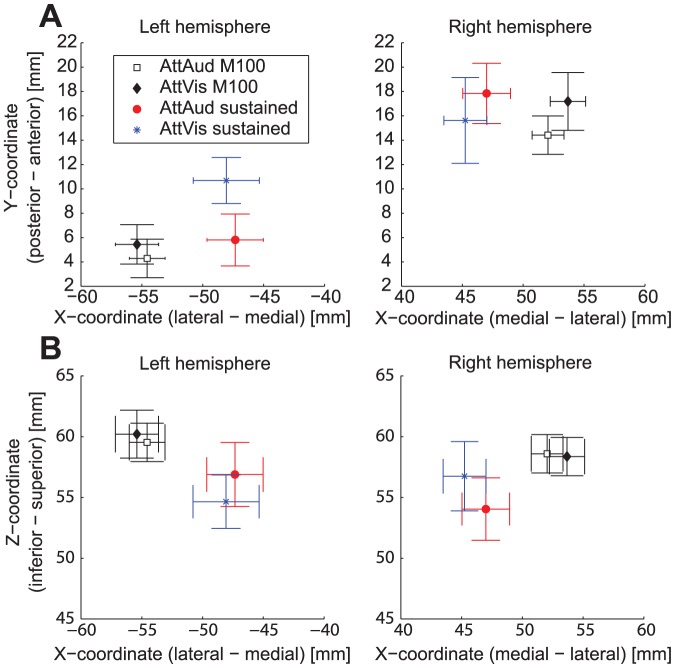
Mean dipole coordinates. Mean (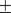
SEM) dipole coordinates for M100 and sustained responses in both conditions in MEG head coordinate system (N = 14). (A) Coordinates in XY plane. (B) Coordinates in XZ plane.

The equivalent current dipoles (ECDs), modeling the sensor-level N100m response and referred to as M100 response in the manuscript, were localized in left and right planum temporale, in superior temporal gyrus (STG) posterior to Heschl's gyrus (HG). Mean dipole locations in stereotactic MNI space in AttAud condition were x = 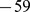 mm, y = 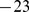 mm, z = 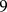 mm for left, and x = 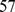 mm, y = 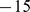 mm, z = 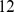 mm for right hemisphere (N = 10, subgroup of subjects with MRI images, see Materials and Methods). Dipole locations for AttVis condition did not differ significantly from those in the auditory condition. The activation foci at M100 latency are visualized using dynamic statistical parametric (dSPM) maps [23] with AttAud condition shown in Figure 2A. Sustained-response ECDs were localized in absolute terms slightly but statistically significantly more medially compared with the M100 (Fig. 3). Figure 2B displays the full time scales of left and right dipole sources.

**Figure 4:**
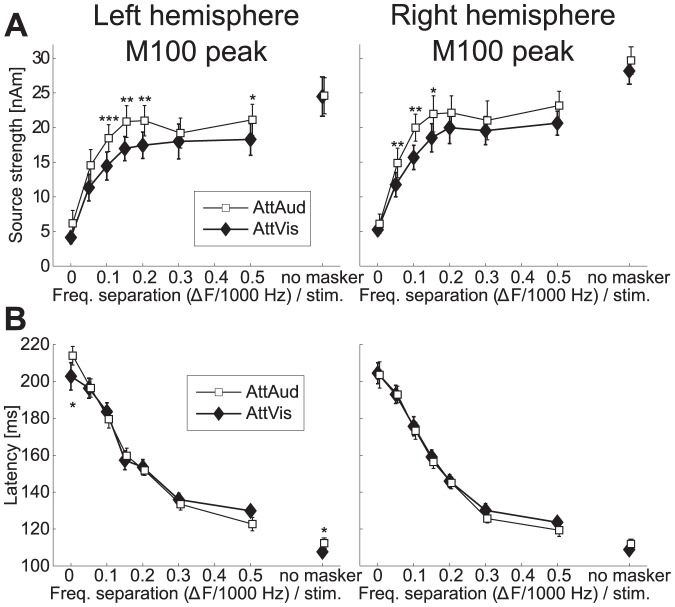
M100 peak amplitudes and latencies. (A) M100 source strengths (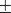
SEM) were clearly modulated by masker type and attention. (B) M100 peak latencies (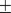
SEM) show strong effect of masker type, nearly doubling in magnitude from the widest notch (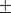
500 Hz, or 0.5 above) to the white noise masker. Attention-induced differences in latency occurred only for the no masker and white noise (0) stimuli in the left hemisphere. Significant changes between AttAud and AttVis are indicated by asterisks (* 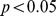, ** 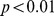, *** 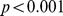).

The M100 peak amplitude was clearly modulated as a function of masker notch width (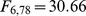, 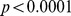, 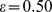), ranging on average from 5.4 nAm (0 Hz masker) to 20.8 nAm (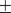
500 Hz masker), thus showing nearly a four-fold increase (Fig. 4A). The source strengths were on average 18% (2.8 nAm) stronger during selective auditory attention (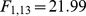, 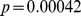). Despite failing to reach statistical significance in the interaction term of the ANOVA, attention effect was not constant in amplitude with different maskers, but showed a clear tendency of being largest with narrow notches (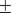
150 Hz,
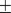
100 Hz, 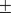
50 Hz). There were no hemispheric effect (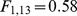, 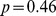) nor significant interaction terms (e.g., MASKERTYPE
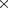
ATTENTION interaction: 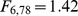, 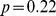, 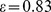). The M100 peak latencies showed a significant dependency on the masker notch width (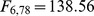, 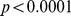, 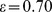), with mean range of 124–206 ms (Fig. 4B).

**Figure 5:**
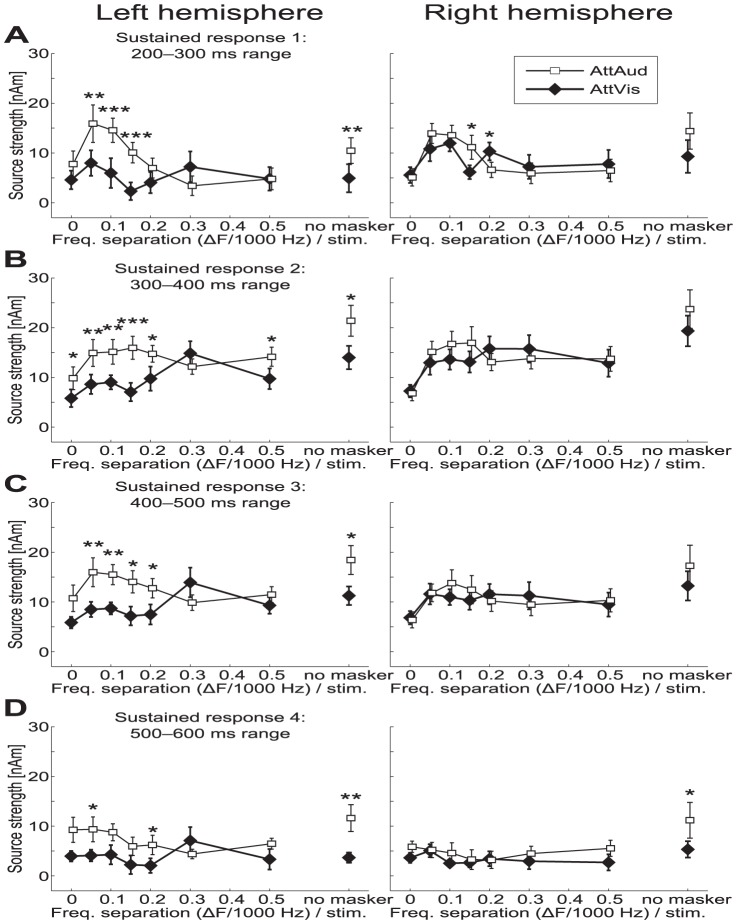
Sustained response amplitudes. Mean evoked activity (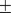
SEM) from (A) 200–300 ms, (B) 300–400 ms, (C) 400–500 ms, and (D) 500–600 ms time range after the sound onset. We selected (B) as the representative time range for the later analysis (see Materials and Methods). Attentional modulation is strongest in (A)–(C) with some of the narrowest notches (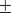
50–200 Hz maskers), suggesting increase in feature specificity within the classical critical band of approximately 160 Hz at 1,000 Hz. Significant changes between AttAud and AttVis are indicated by asterisks (* 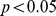, ** 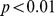, *** 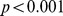).

Sustained responses, defined in the text as 300–400 ms time range from stimulus onset (Fig. 2), showed strong modulations by both masker and attention, with complex patterns of effects (Fig. 5). Sustained responses show the asymmetry of attentional modulation more clearly than the M100 amplitude depicted in Figure 4. Selective attention to sounds enhanced the responses especially in the left hemisphere with notches narrower than 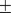
200 Hz, within the critical band. With sustained response, main effects of MASKERTYPE (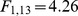, 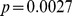, 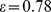) and ATTENTION (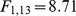, 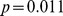) emerged. Importantly, attentional modulation was dependent on the notch width (MASKERTYPE
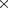
ATTENTION 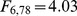, 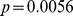, 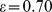), with differential patterns of the attention effect especially with the narrow notches in the left hemisphere (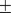
150 Hz,
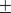
100 Hz, 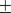
50 Hz; see Fig. 5A–C).

Sustained responses were localized on average 7 mm more medial (see Fig. 3; both left and right hemisphere difference 7 mm, 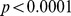; two-tailed 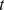-tests) and 4 mm inferior to (left 4 mm, 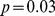; right 3 mm, 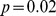; two-tailed 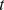-tests) M100 sources in both hemispheres, and 3 mm more anterior (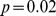; two-tailed 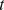-test) in the left hemisphere, which is consistent with earlier findings [24]. There were no statistically significant differences in locations between conditions (AttAud vs. AttVis) in either M100 or sustained response locations.

### Cortically constrained MNE data analysis for auditory cortex

**Figure 6:**
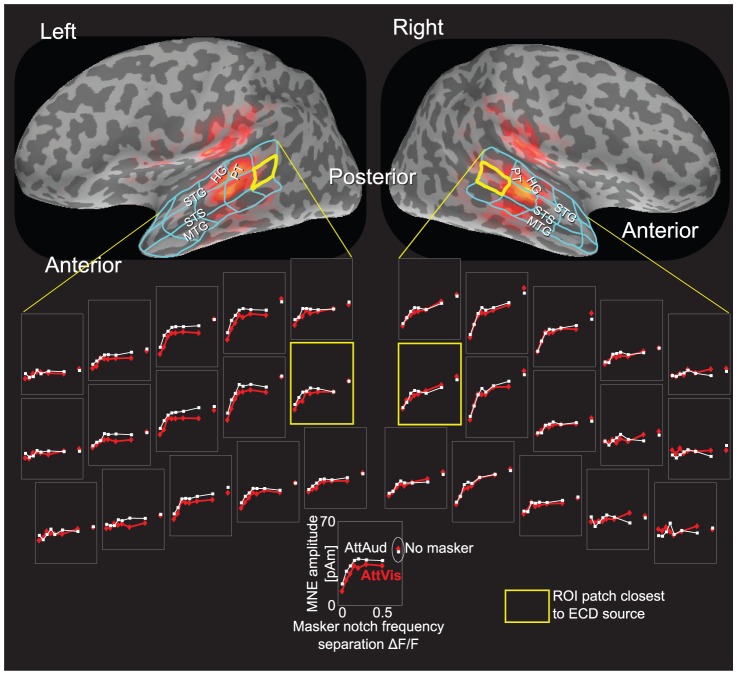
Spatial extent of M100 activity and population-level frequency tuning. Auditory and peri-auditory cortex were divided into roughly equal-sized cortical patches to investigate possible areal differences in frequency tuning and attention effect. The attention effect did not show any significant within- or across-hemisphere asymmetry, but there was a hemispheric interaction effect suggesting asymmetry in the frequency tuning between left and right hemisphere.

In order to better localize the auditory cortex activity, we explored the MEG data using a cortically constrained MNE [25], [26] analysis for the subset of (N = 10) subjects with MRIs. To get an initial assessment of the activity, the whole auditory cortex region of interest (ROI), extending over temporal areas (superior temporal sulcus, including Heschl's gyrus), was selected and the mean amplitude of vertices was taken as the measure. The results from this analysis were in line with the ECD analysis. When auditory cortex area was divided to roughly equal regions (Fig. 6), the obtained MNE data showed main effects of MASKERTYPE (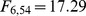, 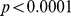, 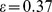), ATTENTION (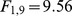, 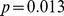) and GRIDPOINT (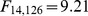, 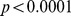,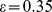), with no hemispheric main effect. Importantly, the hemispheric and spatial dependency of the attention effect was evident as both significant ATTENTION
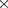
HEMISPHERE interaction (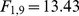, 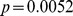) and ATTENTION
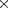
GRIDPOINT interaction term (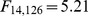, 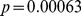, 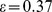). Varying frequency tuning at different grid points, which can be seen as variability of curves in Figure 6, resulted a significant MASKERTYPE
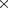
GRIDPOINT interaction (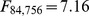, 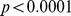, 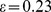).

**Figure 7:**
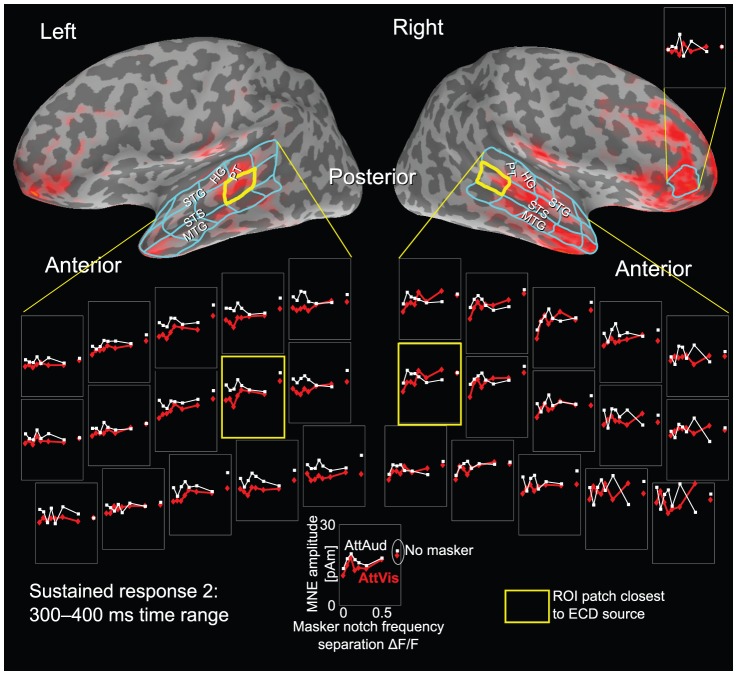
Spatial extent of sustained activity and population-level frequency tuning. Similar to Figure 6, but for the sustained response range showing the most prominent attention effects (300–400 ms). The attention effects are more consistent in the left hemisphere, but show no clear within-hemisphere differences. The data in the right hemisphere are more variable and noisy, showing less consistent masker type specificity in the response magnitude than left hemisphere. Note that due to long latency, the evoked response visualization in the right hemisphere is showing activity already in frontal areas, possibly related to some higher-order cognitive processes not handled in this study. Notably this frontal activity did not show any masker dependency.

Sustained response grid analysis (Fig. 7) using MNE did not show spatial changes due to attention, but revealed a hemispheric effect (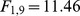, 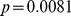). Similar to dipole modeling results, the main effect of attention was significant (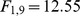, 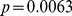) and dependent on masker type (MASKERTYPE
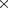
ATTENTION 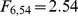, 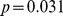, 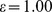).

### Behavioral results

We used sensitivity index 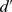 [27] to characterize behavioral performance. This index takes into account false alarms in order to reduce possible response bias and is defined as difference of z-transformed hit rate and false alarm rate, 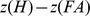. Basically a higher 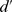 value tells that the subjects perform better in the given task, or the signal is more easily detected.

**Figure 8:**
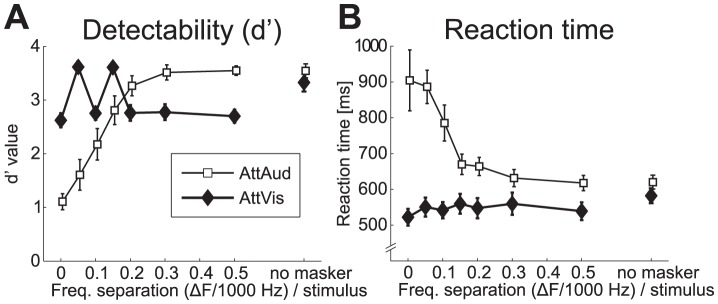
Behavioral data. (A) Detectability (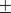
SEM) and (B) reaction time (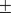
SEM) measures show basically how the task requirements during AttAud condition changed, with gradual increase in 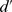 and decrease in RT with the wider notches. AttVis control task was intended to be comparable to AttAud, and 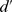 suggests that the difficulty level was well adjusted in comparison with AttAud, thus probably not causing changes in subjects' vigilance level (see Fig. 9).

Behavioral data showed significant main effects of MASKERTYPE (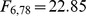, 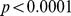, 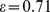; 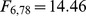, 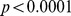, 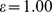) and ATTENTION (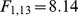, 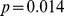; 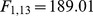, 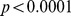) on performance measure 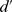 and reaction time (RT), respectively, accompanied with highly significant MASKERTYPE
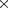
ATTENTION interaction (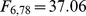, 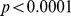, 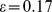; 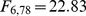, 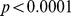, 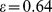). The significant interaction term stems from differential effects of masker type on performance depending on the focus of attention, as can be seen in Figure 8. Basically for the auditory task (AttAud), 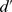 and RT change as a monotonic function of notch width, whereas for visual task (AttVis), 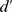 and RT are more uniform with different noise masker types. With no masker, there was no effect of attention on 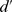, but subjects responded faster during AttVis condition (620 ms vs. 582 ms for AttAud vs. AttVis, 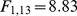, 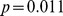). For the RT analysis, 11/224 (4.9%) data were missing with the narrowest notches (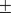
100 Hz, 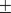
50 Hz, 0 Hz) because some subjects did not detect any deviants. For statistical analysis, these values were imputed by Expectation Maximization (EM) algorithm with a Matlab implementation [28]. Common alternative of mean value imputation was tested as well, and due to small sample size and amount of missing data the results were practically identical to the chosen method. Additionally, it should be noted that in Figure 8, the SEMs were calculated with the real number of data points.

When data from the two attention conditions were tested separately, the main effect of MASKERTYPE for 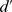 remained significant both for AttAud (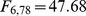, 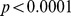, 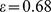, ranging from 1.11 to 3.54) and AttVis conditions (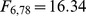, 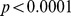, 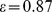, range 2.63–3.61). The main effect of masker for RT was observed only for AttAud condition (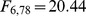, 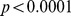, 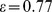). The main effect in 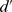 for AttVis condition is due to enhanced performance with 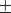
150 Hz and 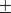
50 Hz maskers (see Fig. 8A). The origin of this effect is unknown, as the video clips including standards and deviants used with those maskers were similarly pseudorandomized from ten precalculated video types (see Materials and Methods) as with other masker types. Further, we did not find a correlation between AttVis task performance and precalculated video type (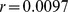, 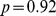 for hit rate, 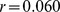, 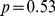 for 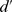), nor was there an order effect due to these masker types always occurring first or last in the whole session. The order effect, however, could in part explain the enhanced performance during AttVis condition with no masker stimulus, as this was always presented first in the session.

### Correlations between behavioral and neurophysiological data

Task performance, measured in 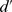, was inversely proportional to RT during AttAud (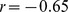, 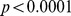) but not with AttVis condition (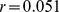, 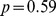). Further, for ECD data, significant correlations were found between 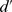 and left (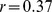, 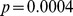) and right (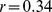, 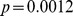) ECD peak M100 amplitude during AttAud condition, and between RT and right ECD amplitude (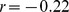, 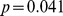). No significant correlations were observed for AttVis data. ECD M100 peak latency turned out to be even more robust correlate for behavioral data, showing significant correlations with AttAud RT (left hemisphere peak latency: 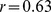, 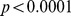; right: 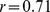, 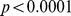) and 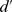 (left: 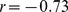, 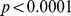; right: 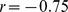, 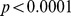). Again, no significant correlations were observed for AttVis data.

**Figure 9:**
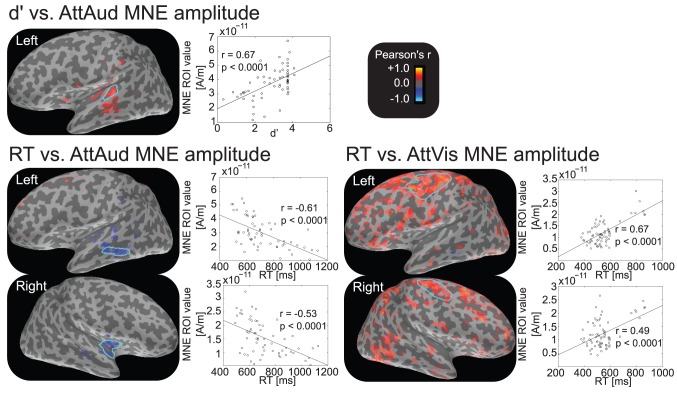
Correlations between behavioral data and MNE activity. The correlations were calculated for each vertex at the M100 peak latency for the per-subject data. The correlations shown on the overlaid cortex are thresholded at 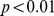. Scatterplots show the mean amplitude of the selected ROI vs. the behavioral measure (across subjects and stimuli). The detectability index 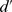 correlated with the vertices showing high M100 amplitude. Reaction time (RT) had a negative correlation in both hemispheres during AttAud condition, whereas significant RT correlations during AttVis condition were probably at least partly artificial due to uneven distribution of data.

When a similar analysis was done for the MNE data, vertex-by-vertex correlation at M100 peak latency with behavioral data confirmed the significant 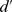 and MNE amplitude dependence during auditory attention. Importantly, after thresholding the data with 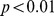, a region of interest was revealed posterior to left HG, in planum temporale, where the MNE showed highest amplitudes and the M100 peak was localized (Fig. 9). With the same threshold, no continuous regions were evident in the right hemisphere, thus further suggesting that left hemisphere activity is of more behavioral relevance in our demanding tone-in-noise detection task. Interestingly, reaction times showed negative correlations in AttAud task with left middle temporal gyrus (MTG) and right insula. For AttVis data, high correlations with 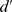 were widespread in posterior frontal and parietal lobes, but no consistent regions were evident for RT. These 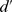 correlations, however, seem to be driven by a number of outliers with high RT and ROI value (see scatterplots in Fig. 9), so they were most probably caused by some subjects who took abnormally long time to answer in the task, showing strong motor cortex activations especially in vicinity of hand and finger area [29]. Nonetheless, in this respect our data were limited in localization accuracy because of unsuited experimental paradigm for motor cortex mapping.

### Time course of frequency tuning and attention effect

With advances in signal processing and MEG shielding technology, reliable averaged MEG responses can be obtained with a significantly smaller number of epochs than the 100–150 typically used for auditory-evoked M100 (see [30]). Here, this advantage was utilized by selectively offline-averaging subsets of data, with the aim of possibly showing refined time course of the attention effect. The data were averaged to 1) first and second presentation of interleaved attention conditions, and 2) seven 30-second segments (with 15-second overlap) of which only non-overlapping segments (0–0:30, 0:30–1:00, …) were used in statistical analysis.

**Figure 10:**
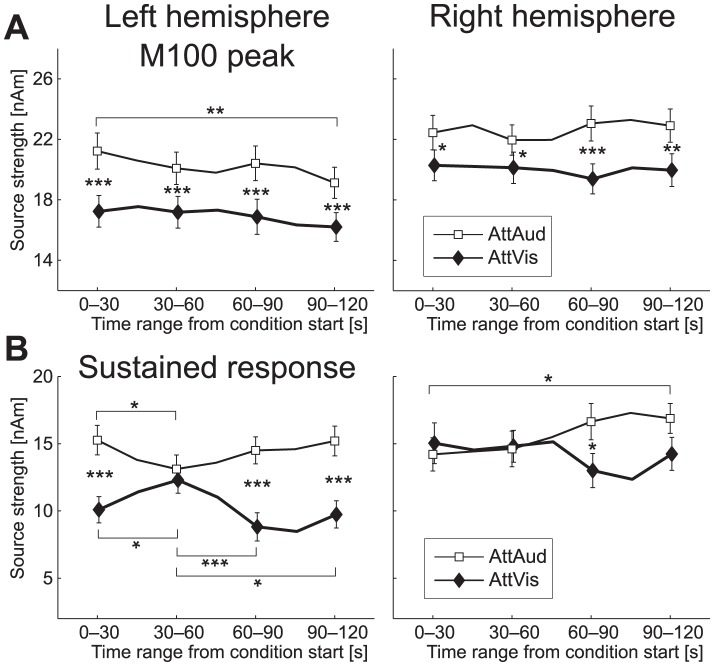
Time course of the attention effect. (A) M100 and (B) sustained response amplitudes (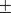
SEM) as a function of time from condition start, averaged across maskers for clarity. The M100 attention effect does not show any dynamics, but the sustained response shows a significant interaction effect with attention and time range from the condition start. Significant differences between AttAud and AttVis are indicated by asterisks between the curves, and significant differences within condition between time ranges are marked either above or below the curves (for AttAud and AttVis, respectively, * 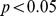, ** 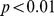, *** 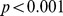).

For M100 response, segment analysis of dipole modeling (ECD) data showed a significant MASKERTYPE
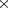
SEGMENT interaction (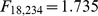, 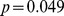, 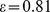), thus tentatively suggesting a differential tuning curve as a function from the start of 2-minute block. This effect was in addition to the same effects of attention and masker reported earlier. There was a trend of stronger M100 amplitudes in the first segment within the 2-minute block, but this effect did not reach significance. This indication of general long-term N100/M100 habituation during the course of stimulus presentation [31] did not reveal anything novel about the time course of the observed attention effect. In fact, the magnitude of the attention effect seemed more or less constant within the block (Fig. 10A).

The same partial-averaging analysis for the sustained responses (Fig. 10B), however, showed that the attention effect was dependent on the time from the two-minute block start. Initially, the attention effect was significant but not very clear in magnitude (0–30 s in Fig. 10B), then nearly vanished after this (30–60 s), and increased towards the end of the block (60–120 s in Fig. 10B). Due to this, ANOVA showed a significant ATTENTION
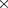
SEGMENT interaction (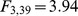, 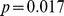, 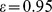), in addition to previously reported masker and attention interaction effects. Further, a significant ATTENTION
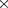
HEMISPHERE effect was found (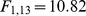, 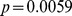), but this may be an artifact of poor signal-to-noise ratio from partial averaging, as selection of different overlapping segments (segments offset by +15 s) removed the effect.

## Discussion

Recent studies have suggested that selective attention enhances both gain and feature selectivity of the human auditory cortex neurons [18], [19]. Here, we show that M100 was modulated bilaterally in such a fashion that most robust attentional enhancements were observed during intermediate masker notch widths (Fig. 4). The longer-latency sustained response, occurring at 300–400 ms range after sound onset was, in turn, even more robustly enhanced by attention in the left hemisphere (Fig. 5B). This enhancement was most prominent with narrow notches within the critical band (i.e., with narrower notches than in case of M100). This finding tentatively suggests that there could be neuronal population-level receptive field tuning that facilitates perception of target sounds embedded in noise especially at the latency of the sustained response.

The attentional modulation of the M100 appeared to occur right after switching of the auditory task, thus supporting the recent findings of Ahveninen et al. [7]. Furthermore, as a novel finding, the sustained response modulation was more dynamic as there was a dependency on the time elapsed from the beginning of the block (Fig. 10), suggesting that differential neural mechanisms underlie these effects. While there were no differences in left and right hemisphere response magnitudes, activity in the left auditory cortical areas exhibited more pronounced correlations with behavioral measures of performance (see Results and Fig. 9), thus supporting previous findings [7]. These results revealed that a strong attentional demand induces changes almost instantaneously, with earlier modulation of the neural population generating the M100 response in both left and right hemisphere posterior secondary auditory areas. After the initial onset response, sustained response at a somewhat later latency showed clear enhancement in sound-feature selectivity in more medial regions of the left hemisphere.

Our current results are in line with earlier studies showing attentional modulation of transient 100-ms onset responses with concurrent masking paradigm [7], [18], [19], [32]–[35]. Naturally, we cannot exclude a possible earlier-latency effect, since attentional modulation can be seen at brainstem [36], [37], and even lower level (see [12]). We could not, however, reliably quantify the 50-ms response due to poor signal-to-noise with the narrowest notches (see Fig. 2B). Similar to a recent MEG study [35], we failed to observe a significant masker
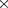
attention interaction in M100 amplitude, however, given that the significant effects were observed for intermediate notch widths it is possible that the attentional enhancement of M100 reflected a combination of gain and receptive field tuning effects in the present study (see Fig. 4A). Further, an interaction with masker, attention, and hemisphere was evident in the sustained response (Fig. 5), which was significantly modulated especially in the left hemisphere, showing most prominent attention effects with narrow notches (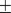
50 Hz–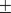
200 Hz), but not with the white noise masker. This possibly reflects different feature specificity [38] and response properties [39]–[41] of distinct M100 and sustained response generators.

The sustained response [24], [42] or post-M100 response increase in sound-feature specificity [43] and enhancement during selective attention have been shown before [44]–[46]. To our knowledge this is the first notched-noise masking paradigm study to report significant masker dependent changes in the sustained response magnitude. This long-latency evoked response has sometimes been referred to as processing negativity (PN) [47] and can vary for instance as a function of task difficulty [48]. However, processing negativity or similar separate process cannot alone explain the current sustained response results unless it is defined to possess feature specificity besides task demand relations. Further, there is evidence from intracranial studies in humans that support the notion that N100/M100 and longer-latency components are directly enhanced during attention [49], [50] instead of supplemental attention-induced activity explaining the response enhancement.

Our results of auditory-evoked M100 and sustained response modulation during a high-load task suggest that selective attention enhances activity in the secondary auditory areas, close to sites where the M100 and sustained responses are localized. M100 is primarily generated posterior to the primary auditory cortex in HG, in posterior supratemporal plane [51], [52]. Unlike in our previous EEG study [18], we failed to observe attentional enhancement of M100 with the no-masker sound. As a potential explanation for this, there are auditory selective attention studies where MEG and EEG have been recorded simultaneously, and in such studies MEG has been noted to be less sensitive to M100 enhancement during selective attention than EEG [53], which could be due to radial sources (that MEG is insensitive to [54], [55]) contributing to the N100 attentional enhancement recorded with EEG. Thus, EEG and MEG results can differ from each other, even if recorded simultaneously [56].

In the present study, the attentional enhancement of sound feature selectivity was more clear for the sustained than the M100 response. N100/M100 response is related to sound onset or any transient change in auditory environment [57]–[60], whereas later components can be more sensitive to bottom-up sound features and top-down modulation [61]. This is possibly related to differential mechanisms in initial enhancement of relevant and later-latency suppression of non-relevant sounds [10], [11]. The dynamics of the attentional effect of both the M100 and sustained response being highest at small and intermediate notches (see Figs. 4 and 5) can be related to lateral inhibition [62] suppressing neural populations differently when attention is focused to sounds compared to baseline (see also [18]). Task difficulty variability (Fig. 8) with notches below 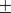
200 Hz can play a role in the presently observed sustained response modulation (Fig. 5), as previous studies have shown that there is an interaction between task difficulty and magnitude of the inhibitory effect [10]. In addition, differences in the temporal dynamics of these two effects (Fig. 10) suggest that attentional modulation of M100 and sustained response might indeed have different underlying mechanisms.

In this study, we recorded population-level MEG responses, which are generated mostly by post-synaptic current summation when thousands of neurons accumulate post-synaptic potentials in synchrony [54]. Thus, caution must be exercised when speculating about single-neuron level mechanisms. Our results show similar tendencies of attentional modulation that animal models have shown at the level of single-neuron receptive fields [63]–[65], where enhancements at the target frequency during task performance are often accompanied by suppression at nearby frequencies, thus increasing the detectability of the target sound. Notably, in these studies, the receptive fields have been quantified by correlating neuronal spiking with spectrotemporal properties of specific type of ongoing background auditory stimulation; thus in these animal models the observed attentional tuning effects reflect more changes in sustained rather than onset responses.

The critical band or equivalent rectangular bandwidth (ERB) at 1000 Hz has been estimated to be 162 Hz [66], [67]. Here, the range of which M100 amplitude was modulated by notch width was 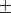
150–200 Hz (Fig. 4A), and our behavioral data suggests an ERB value of between 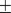
200–300 Hz (Fig. 8) where 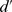 in AttAud condition reaches its plateau. These are well in line with the classical ERB estimate and recent MEG studies [68], [69], and slightly lower than ERB of 248 Hz that was estimated in a previous MEG study using a highly similar masking paradigm [70]. Interestingly, the range of notches where attention modulated the M100 responses was partly beyond the critical band, whereas the sustained response amplitude modulation showed strongest effects within the classical ERB, with notches below 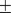
200 Hz (Fig. 5).

In the present study, we contrasted data from two conditions with identical stimuli, and attention was directed to either sounds or visual flankers. Because we omitted passive condition due to problems in controlling the arousal of the subject and in order to keep the experimental time within reasonable limits, it could be argued that the differences between conditions arose from suppression of auditory-evoked response due to visual task processing. However, recent studies have shown that a concurrent visual task with similar difficulty level, irrelevant to sound presentation, does not have an effect on auditory-evoked MEG responses as compared to a passive baseline [30]. Further, as the visual task difficulty level was kept constant during different masker types, the possible cross-modal visual task-induced effect should be constant with different noise maskers (although there is some variation in 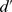 but not in RT, see Fig. 8). Thus, taken together, we can say relatively confidently that the observed effects arose from partly frequency-specific [71] selective attentional enhancements of relevant or suppression of irrelevant sounds during auditory attention, and were not caused by a general suppression induced by the visual task.

## Materials and Methods

### Subjects

Altogether fourteen healthy subjects participated in the study (age 21–46, mean age 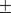 S.D. 29.3
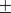
6.2, 10 males). One of the subjects was native English speaker, one a native Russian speaker, and the rest were native Finnish speakers. All subjects had normal hearing and normal or corrected-to-normal sight, and all but two were right-handed. All subjects were university students or staff, and were not paid for participation. A subset of 10 subjects (7 males) was used in minimum norm estimate (MNE) analysis as their cortical surface reconstructions from whole-head anatomical MRI images were readily available.

### Ethics Statement

All subjects signed a written informed consent before the study. The entire study had a prior approval by the Ethics Committee of the Hospital District of Helsinki and Uusimaa, Finland, and the experiment was run in accordance with the Helsinki Declaration.

### Experimental setup

Stimulus and experimental setup is depicted in Figure 1. The stimulus setup is nearly identical to our earlier EEG study [18], the main differences being the inclusion of more notch widths, only one auditory task (AttAud), and visual control task (AttVis) instead of passive baseline. In the current experiment, auditory and visual stimuli were identical during both AttAud and AttVis conditions, only the masker sound was changed in each block. The subjects were instructed to follow the instructions on the screen and to respond as accurately as possible. They were cued to attend either modality at the beginning of the 8-minute block, and at each condition change every two minutes (see Fig. 1C) by text instructions shown on the screen. The evoked responses were discarded during these presentations of task instructions. The order of the AttAud and AttVis conditions were pseudo-randomized. The target response was detected using an optical response device, where the subjects lift their right index finger to answer. The frequency of the deviants for both auditory and visual stimuli were adjusted during the design of the experiment based on several psychoacoustic sessions. The aim was to keep the attentional demand high and to avoid a ceiling effect in the course of the experiment by using stimuli only slightly above the threshold.

Auditory stimuli were constructed at 16-bit, 48-kHz with Matlab (R14, MathWorks Inc., Natick, MA, USA). The standard stimulus for which evoked responses were recorded was 300-ms, 1000-Hz tone with 5-ms onset and offset ramps. The 1020 Hz target tone (identical in other aspects to the standard) was presented at random intervals 10% of the time. The continuous masker sounds (16-bit, 48-kHz) were created in Matlab by filtering in frequency domain 10-minute Gaussian white noise with symmetrical stopbands or notches around 1000 Hz. For example, for 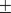
150 Hz masker, stopband of 850—q1150 Hz was used. Thus, due to logarithmic nature of frequency perception [72], lower range of the notched-noise masker contributes more to the frequency masking. The tones were presented with a mean onset-to-onset interval of two seconds (range 1800–2200 ms). The slow rate was used in order to obtain good enough MEG signal-to-noise with the narrowest notches and the white noise masker.

Visual stimuli consisted of Gabor patches or flankers with an identical orientation and predefined spatial frequency. Similar to the auditory stimulus, 10% of the flankers were deviants with a slightly higher spatial frequency. The still frames were concatenated to a 5 FPS video file using Xvid encoder (http://www.xvid.org) with parameters to maximize image quality. Each flanker was presented for 400 ms (2 frames) and the onset-to-onset interstimulus interval for each flanker was constantly 1.8 seconds (9 frames). Ten video clips were created with a different presentation order, and they were presented in random order in each block so that subjects could not learn the presentation pattern.

The sounds were presented through a high-quality 60×60 cm panel speaker (Panphonics SSH-SQW sound shower, Panphonics, Espoo, Finland), which is able to reproduce frequency response of 400 Hz–16 kHz (−6 dB/oct.). The speaker was mounted on the wall of the shielded room in front of the MEG device, directed to the subject's head, so a natural binaural perception was possible. The visual stimuli were presented through a back-projector screen located in front of the subject at a distance of 1.5 m. As the auditory and visual stimuli were presented asynchronously in their own streams with a different rate, they could not be fused to an audiovisual object. Stimulus presentation was controlled by a computer running Presentation software (v12.0, Neurobehavioral Systems Inc., Albany, CA, USA).

Before the MEG acquisition began, the subject was introduced to the task and stimuli outside of the MEG shielded room. The subject then entered the room and was seated comfortably under the MEG device. After this, individual 50% hearing threshold was estimated using an up-down procedure [73], where the level of the white noise masker was changed so that the embedded tone-in-noise was barely audible. Following this threshold estimate, the no-masker condition (with only tones playing) was presented to the subject, lasting 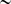
8 minutes. Finally, 8-minute blocks spanning all masker sounds (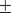
500 Hz, 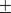
300 Hz, 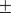
200 Hz, 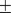
150 Hz, 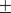
100 Hz, 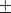
50 Hz, 0 Hz) were presented in randomized order, counterbalanced across subjects.

### MEG acquisition

Magnetoencephalography (MEG) was measured in a magnetically shielded room located in the O.V. Lounasmaa Laboratory of Aalto University using a whole-head neuromagnetometer (Vectorview, Elekta Neuromag Oy, Helsinki, Finland) with 306 channels. The device has 102 sensor elements, each with two orthogonal gradiometers and a magnetometer. The continuous MEG data was recorded at 2000 Hz, with a passband of 0.1–650 Hz, except for the additional microphone channel, where lowpass filtering was disabled in order to detect onset of tones and to verify that the auditory stimulus presentation was accurate with a minimal jitter. To detect eye blinks and movements, one electro-oculogram (EOG) channel was recorded with the electrodes placed below and on the outer canthus of the left eye. Auditory-evoked responses to both standard and deviant tones were averaged. MEG was online-averaged to assess the initial quality of the data and to verify that at least 100 artifact-free epochs were obtained, but the actual analyses were made with offline-averaged data. In the data analysis, offline-averaged −200–800 ms time-locked epochs exceeding 3000 fT/cm (in any gradiometer channel), 400 fT (in any magnetometer channel) or 150 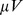 (in EOG channel) were rejected for possibly containing extracerebral activity. Prestimulus baseline of 200 ms was used to remove DC offset, and 40 Hz lowpass filter was used during averaging. Before moving the subject to the shielded room, 3D locations of left and right preauricular points and nasion, four head-position indicator (HPI) coils, and a number of extra points from the scalp were digitized to obtain a right-handed head-coordinate frame that was used later in dipole localization and alignment with cortical surface reconstruction for MNE analysis. The HPI coil locations were used in estimating head position at the beginning of each recording block.

### MEG data analysis

**Figure 11:**
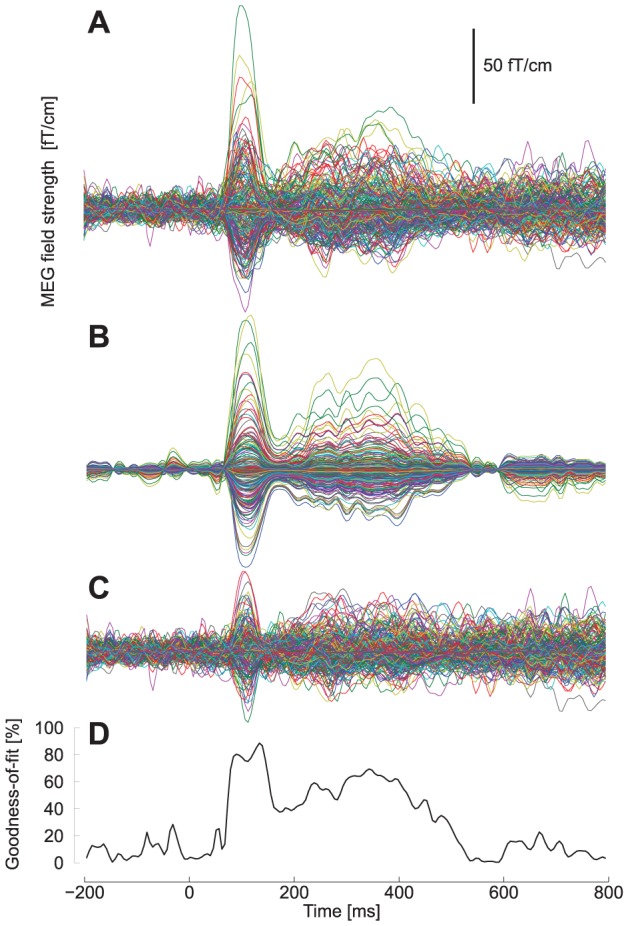
Dipole modeling results for a representative subject. Butterfly plot showing MEG fields from all sensors with (A) real data from one subject with AttAud condition and no masker, (B) two-dipole model fitted at around N100m peak latency, (C) residual field (data vs. model). (D) The N100m response is well explained by the dipole model, goodness-of fit curve peaked at 80–90%.

Two equivalent current dipoles (ECD) were used to model the current sources of the MEG signals at around N100m peak latency for both hemispheres using a spherical head model and gradiometer data [54]. The data from ECD modeling are referred to as M100 in the manuscript to dissociate them from the sensor-space N100/N100m effect. For each subject, ECDs were estimated using Neuromag (Elekta Neuromag Oy, Helsinki, Finland) Xfit software for the no masker stimulus, as this stimulus provided the best signal-to-noise quality. ECD model explained typically over 80% of the field variability at the N100m peak latency (Fig. 11). These dipoles were used to project the data at different maskers using a fixed dipole approach, assuming that signal sources stay identical with the inclusion of masker. This assumption was verified in a selection of subjects by comparing 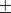
500 Hz dipole fits to no masker dipole fits, and results support the findings of previous studies [19] that signal sources are practically unaltered with the addition of a continuous masker sound. As source locations from AttAud and AttVis conditions were practically identical and did not differ (see Fig. 3), the data from both conditions were projected using the AttAud fixed dipole.

Sustained responses were modeled, similar to M100, by two per-subject equivalent current dipoles at most prominent part of the sustained field (at around 330–380 ms, before the offset response at around 400 ms). The source locations were close to M100 dipole source in supratemporal plane, but especially the AttAud sustained response sources were localized on average 7 mm more medial than the corresponding M100 sources (see Results and Fig. 3; total distance between sources 14 mm). Due to this, sustained responses were quantified by projecting the data through the two fixed sustained response dipoles and taking the source strength from the corresponding source waveforms, although the M100 dipole was able to capture most of the activity at this latency, as shown in Figure 2.

ECD data were analyzed using analysis of variance (ANOVA) with factors MASKERTYPE (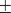
500 Hz, 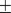
300 Hz, 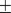
200 Hz, 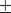
150 Hz, 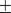
100 Hz, 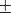
50 Hz, 0 Hz), ATTENTION (AttAud, AttVis) and HEMISPHERE (left, right). For MNE auditory cortex grid analysis an additional GRIDPOINT (1, …, 15) factor was used. The reported 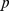-values are Huynh-Feldt corrected with 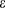 measure documented where appropriate, but original, uncorrected degrees of freedom are reported. The no masker data were tested separately because (1) auditory stimulus was so clearly different from the masker conditions, and (2) no masker was always presented at the beginning of the experiment, thus probably causing an additional bias in the data.

M100 peak measure was quantified as the mean value of peak latency 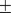
10 ms time range. Peak detection was done using a semiautomatic algorithm. Notably, in this case, the M100 response latency varied from 100 to over 200 ms with white noise masker (see Fig. 4B), thus a term “onset response” to sound would better describe the measure that was used rather than categorization of deflections by their typical latency. For simplicity, we will use the M100 term throughout the manuscript. Sustained response was defined as mean amplitude of 100-ms range at 300–400 after the sound onset (see Fig. 2B).

MNE data analysis was conducted using MNE suite (v2.7.0, http://www.nmr.mgh.harvard.edu/martinos/userInfo/data/sofMNE.php) for a subgroup of N = 10 subjects for which MRI images were available, and thus in whom surface reconstructions were possible. The inflated surface reconstructions [21], [22] were done using FreeSurfer (v4.5.0, http://surfer.nmr.mgh.harvard.edu/) software.

### Behavioral data analysis

For behavioral data, initially a two-way ANOVA with factors MASKERTYPE and ATTENTION was used. Additional post-hoc contrasts were calculated with AttAud and AttVis conditions separated. All statistical analyses were done in SPSS (version 15.0 for Windows, SPSS Inc., Chicago, IL, USA).
